# A Vegetarian Diet Is a Major Determinant of Gut Microbiota Composition in Early Pregnancy

**DOI:** 10.3390/nu10070890

**Published:** 2018-07-12

**Authors:** Helen L. Barrett, Luisa F. Gomez-Arango, Shelley A. Wilkinson, H. David McIntyre, Leonie K. Callaway, Mark Morrison, Marloes Dekker Nitert

**Affiliations:** 1 Department of Endocrinology, Mater Health, South Brisbane, QLD 4101, Australia; shelley.wilkinson@mater.org.au; 2 Mater Research Institute, University of Queensland, South Brisbane, QLD 4101, Australia; david.mcintyre@mater.org.au; 3 School of Chemistry and Molecular Biosciences, The University of Queensland, St Lucia, QLD 4072, Australia; luisa.gomezarango@uq.net.au (L.F.G.-A.); m.dekker@uq.edu.au (M.D.N.); 4 Department of Dietetics and Foodservices, Mater Health, South Brisbane, QLD 4101, Australia; 5 Department of Obstetric Medicine, Royal Brisbane and Women’s Hospital, Herston, QLD 4059, Australia; Leonie.Callaway@health.qld.gov.au; 6 The University of Queensland Centre for Clinical Research, Faculty of Medicine, Herston, QLD 4059, Australia; 7 Diamantina Institute, Faculty of Medicine, The University of Queensland, Translational Research Institute, Woolloongabba, QLD 4102, Australia; m.morrison1@uq.edu.au

**Keywords:** microbiota, pregnancy, vegetarian

## Abstract

The composition of the gut microbiota can be influenced by dietary composition. In pregnancy, the maternal gut microbiome has associations with maternal and infant metabolic status. There is little known regarding the impact of a vegetarian diet in pregnancy on maternal gut microbiota. This study explored the gut microbiota profile in women who were vegetarian or omnivorous in early gestation. Women were selected from participants in the Study of PRobiotics IN Gestational diabetes (SPRING) randomised controlled trial. Nine women identified as vegetarians were matched to omnivorous women in a 1:2 ratio. Microbiota analyses were performed using 16S rRNA gene amplicon sequencing and analysed using the Quantitative Insights Into Microbial Ecology (QIIME) and Calypso software tools. There was no difference in alpha diversity, but beta diversity was slightly reduced in vegetarians. There were differences seen in the relative abundance of several genera in those on a vegetarian diet, specifically a reduction in *Collinsella*, *Holdemania*, and increases in the relative abundances of *Roseburia* and *Lachnospiraceae*. In this sub-analysis of gut microbiota from women in early pregnancy, a vegetarian as compared to omnivorous diet, was associated with a different gut microbiome, with features suggesting alterations in fermentation end products from a mixed acid fermentation towards more acetate/butyrate.

## 1. Introduction

The composition of the gut microbiota has been associated with host metabolic status and health and is affected by dietary composition [1]. The impact of vegetarian diets on the gut microbiota in adults has been examined in observational and interventional trials. In a cross-sectional study of 101 adults in Italy, greater richness, and increased abundance of the phylum *Bacteroidetes* was observed in the vegetarian compared to vegan and omnivore groups [2]. Another cross-sectional cohort examined 268 non-diabetic participants who were strict vegetarians, lacto-ovo vegetarians and omnivores [3]. The abundance of the phylum *Firmicutes* was lower and *Bacteroidetes* was higher in strict vegetarians, with the genus *Prevotella* being increased amongst other changes [3]. A prospective study addressed the changes in response to the adoption of a lacto-ovo-vegetarian diet for three months in a group of 15 omnivores, in comparison to continuous omnivores (*n* = 7) and long term vegetarians (*n* = 7) [4]. Adoption of a vegetarian diet did not change individual diversity of the gut microbiota (alpha diversity) but caused a decrease in the diversity (beta diversity) in the group of people who changed to a vegetarian diet. In addition, increases in the abundance of the genera *Roseburia* and *Ruminococcus*, which are known to be involved in digestion of plant polysaccharides, were observed. Those individuals who adhered to a long term vegetarian diet also showed enrichment in the genera *Haemophilus*, *Neisseria*, *Aggregatibacter*, and *Veillonella* [4].

Plant-based diets are thought to be beneficial in the prevention and control of type 2 diabetes and in reducing cardiovascular risk factors [5]. There is limited evidence that this may be mediated in part through modulation of gut microbiota. When 6 obese participants with type 2 diabetes (*n* = 4) and/or hypertension (*n* = 2) were assigned to a vegetarian diet for 1 month, a reduction in body weight, and improvement in metabolic markers was observed. There was also a reduction in the *Firmicutes* to *Bacteroidetes* ratio in the gut microbiota, with decrease in *Enterobacteriaceae* and increase in *Clostridium* species and *Bacteroides fragilis* [6].

The composition of the gut microbiota is generally reported to be affected by pregnancy [7]. There are no studies the authors are aware of that have investigated the composition of the gut microbiota in women maintaining a vegetarian diet during pregnancy. This cohort from the Study of PRobiotics IN Gestational diabetes (SPRING) [8] randomised controlled trial supplementing overweight or obese women with a probiotic offers the opportunity to examine the microbiota of women in early pregnancy, prior to supplementation. The analysis described below aims to analyse the association between dietary patterns on maternal gut microbiota in early pregnancy in this well characterised cohort with detailed dietary and metabolic data.

## 2. Materials and Methods

### 2.1. Participants

Women were selected from participants in the Study of PRobiotics IN Gestational diabetes (SPRING; ANZCTR 12611001208998) randomised controlled trial [8] who completed the food frequency questionnaire and supplied a stool sample at baseline (<16 weeks gestation). The study was approved by the human research ethics committee of the Royal Brisbane and Women’s Hospital (HREC/11/QRBW/467) and The University of Queensland (201200080) and all participants provided informed written consent. The study enrolled only overweight or obese women. Nine women identified as vegetarians and they were matched to omnivorous women in a 1:2 ratio. The matching was performed on maternal body mass index (BMI) at baseline, total energy intake at 16 weeks gestation and the future development of gestational diabetes mellitus. Each woman completed the Victoria Cancer Council Food Frequency Questionnaire (Version DQES V2.0) with instruction to include only dietary information since the beginning of pregnancy. This dietary information was analysed for macronutrient and fatty acid content. Women also provided a self-collected stool sample, which was kept cold until storage at −80 °C prior to faecal DNA isolation. In addition, fasting blood samples; anthropometric measurements and medical and obstetric history were obtained from all participants. 

### 2.2. Faecal DNA Extraction

Stool samples were thawed at 4 °C prior to subsampling ~250 mg stool for DNA extraction using the repeated bead beating and column (RBB + C) protocol and Qiagen AllPrep columns for DNA purification [9,10]. The faecal subsample was mixed with the RBB + C lysis buffer and sterile zirconia beads (0.1 and 0.5 mm diameter) and homogenised using a TissueLyser II (Qiagen, Chadstone, VIC, Australia) for 3 min at 30 Hz. DNA quantity and quality were analysed using the Nanodrop ND 1000 spectrophotometer (NanoDrop Technologies, Thermo Scientific, Scoresby, VIC, Australia) system.

### 2.3. 16S rRNA Sequencing

The V6-V8 hypervariable regions of the bacterial 16S rRNA gene in stool DNA extracts were PCR amplified using the 926F forward (5′-TCG TCG GCA GCG TCA GAT GTG TAT AAG AGA CAG AAA CTY AAA KGA ATT GRC GG-3′) and 1392R reverse (5′-GTC TCG TGG GCT CGG AGA TGT GTA TAA GAG ACA GAC GGG CGG TGW GTR C-3′) primers. Positive (*E. coli* JM109 DNA) and negative (deionised sterile water) controls were included in each PCR run. The PCR products were barcoded with the Nextera XT V2 index kit Sets A and B (Illumina, San Diego, CA, USA), and purified with the AMPure XP bead system (Illumina, San Diego, CA, USA). Sequencing libraries were prepared after quantification, normalisation and pooling of the barcoded DNA and sequenced on the Illumina MiSeq platform (Illumina, San Diego, CA, USA) at the Australian Centre for Ecogenomics at The University of Queensland. Forward and reverse sequences were joined and de-multiplexed using the Quantitative Insights Into Microbial Ecology (QIIME) v1.9.1 analysis tool [11]. The open reference operational taxonomic unit (OTU) picking method was used for taxonomic assignments via the Greengenes reference database, with a pairwise identity threshold of 97%. Any OTUs present in the negative controls were removed from the analysis as were OTUs with a relative abundance of <0.0001. The OTU table was rarefied to 3000 sequences/sample prior to downstream analysis. No samples were removed in the rarefaction step.

### 2.4. Statistical Analysis

The abundances of the bacteria were not normally distributed and are therefore presented as median and interquartile range (IQR). Non-parametric statistical methods were used throughout the study with a *p* value cut-off of <0.05 for statistical significance. The online Calypso software tool [12] was used to analyse the sample profiles, presenting primarily the results at the family and genus level of taxonomic assignments. The Chao1, Shannon, ACE, Evenness, Richness and Simpson indices were used for comparison of within sample (alpha) diversity. Between sample (beta) diversity variation was compared using the permutation multivariate analysis of variance (PERMANOVA) in the Adonis tool. Clustering of the samples was analysed by canonical correspondence analysis. Network analysis was performed to identify positive and negative correlations between bacterial taxa for both the vegetarian and omnivorous groups. Genera associated with a vegetarian or omnivorous diet were identified using Spearman’s rho correlation coefficients with 1000-fold permutations. The results are reported as significant if the false discovery rate (FDR) was <0.05, with the degree of colouring of the nodes reflecting the level of significance of association with either diet type. The size of the nodes indicates the abundance of the genus. The predicted functions of the microbiome were analysed with the PICRUSt software tool [13]. LefSe (Linear discriminant analysis (LDA) effect size) analysis was carried out to identify genera discriminating between the groups [14].

## 3. Results

### 3.1. Study Participants

For this sub-analysis, the vegetarian group was selected from the women who supplied a faecal sample and dietary information at 16 weeks gestation. Only nine women followed a vegetarian diet. Each of these women was matched with two women who were similar in BMI, future gestational diabetes status and overall energy intake (Table 1).

This ensured that the groups were more even in size rather than comparing the vegetarian women to all omnivorous women. The overall reported energy intake was low, suggesting possible underreporting, but not different between groups. Women on a vegetarian diet had similar blood pressure and slightly higher fasting blood glucose than women on an omnivorous diet. However, their overall future gestational diabetes mellitus (GDM) rates were not different from the overall Study of PRobiotics IN Gestational diabetes (SPRING) population (11.1% vs. 15.3%, *p* = 1). Women eating a vegetarian diet had significantly lower intake of protein, sugars and saturated fat but higher intake of polyunsaturated fat (PUFA) (Table 2). They also had higher levels of dietary linoleic acid (ω-6), and lower levels of arachidonic acid, eicosapentaenoic acid (EPA) and docosahexaenoic acid (DHA).

### 3.2. Comparison of Overall Gut Microbiota Composition

**Figure 1:**
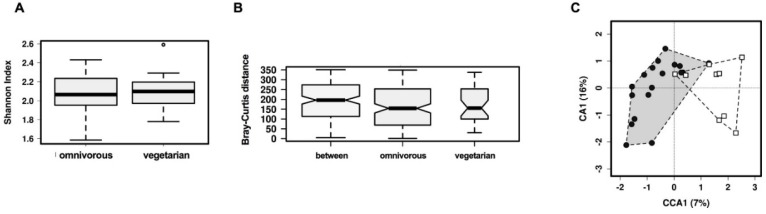
Measures of diversity, comparing vegetarian to omnivorous participants. (**A**) Shannon Index, Box plots show the 2.5th and 97.5th percentile with a line at the median; (**B**) Bray–Curtis Distance, Box plots show the 2.5th and 97.5th percentile with a line at the median; (**C**) Canonical correspondence analysis at the genus level according to diet. The percentage of variation is shown. This hypothesis-driven technique suggests that diet significantly affects gut microbiota composition (*p* = 0.017). Black circles, omnivorous diet; white squares, vegetarian diet.

While there was no significant differences between the groups in terms of their alpha (within sample) diversity metrics for any of the indices analysed at phylum (Figure S1), genus (Shannon, *p* = 0.56, Figure 1A and Figure S2) and OTU level (Figure S3), the beta diversity between groups was significantly different at genus and OTU level but not at phylum (genus level R = 0.16; *p* = 0.046, Figure 1B; OTU level R = 0.17; *p* = 0.041, Figure S4A) as was the canonical correspondence analysis of these profiles (genus level *p* = 0.021, Figure 1C; OTU level *p* = 0.017, Figure S4B).

**Figure 2:**
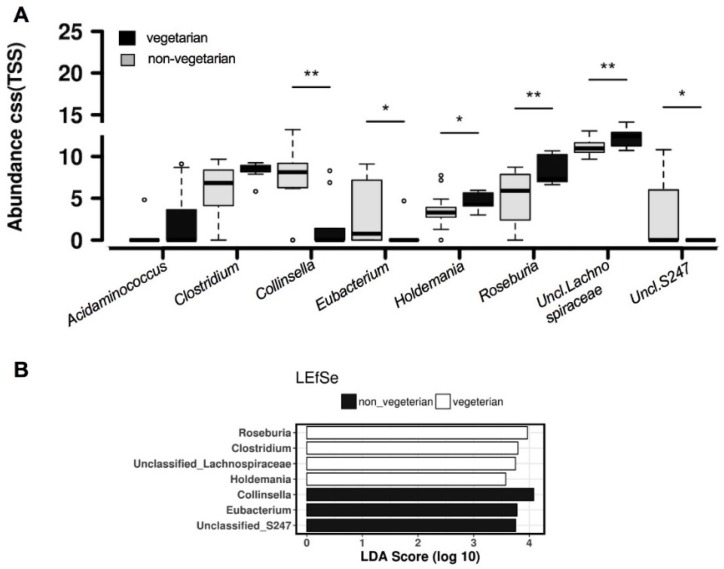
Linear discriminant analysis (LDA) effect size (LEfSe) analysis, (**A**) Abundance, Box plots show the 2.5th and 97.5th percentile with a line at the median; (**B**) LDA Score. *, *p* < 0.05; **, *p* < 0.01; º, individual values <2.5th or >95th percentile.

When comparing the composition of the gut microbiota between the groups, there were no differences in abundance at phylum level. At genus level, women consuming a vegetarian diet possessed significantly lower abundances of *Collinsella* (*p* = 0.0059), *Holdemania* (*p* = 0.025), *Unclassified S247* (*p* = 0.039), and *Eubacterium* (*p* = 0.041), but had significantly higher relative abundances of *Roseburia* (*p* = 0.0064) and *Unclassified Lachnospiraceae* (*p* = 0.0087) and the relative abundances of *Clostridium* and *Acidaminococcus* approached statistical significance (*p* = 0.060 and *p =* 0.061, respectively, Figure 2A). Similarly, the LEfSe analysis identified the genera *Roseburia*, *Clostridium*, *Uncl. Lachnospiraceae* and *Holdemania* as biomarker genera for a vegetarian diet whereas *Collinsella*, *Eubacterium* and *Uncl. S247* as biomarker genera for an omnivorous diet (Figure 2B). These results were in line with the comparisons at family (Figure S5) and OTU level (Figures S6 and S7), which also indicate that members of these genera are altered in a similar direction by a vegetarian diet.

### 3.3. Network Analysis

**Figure 3:**
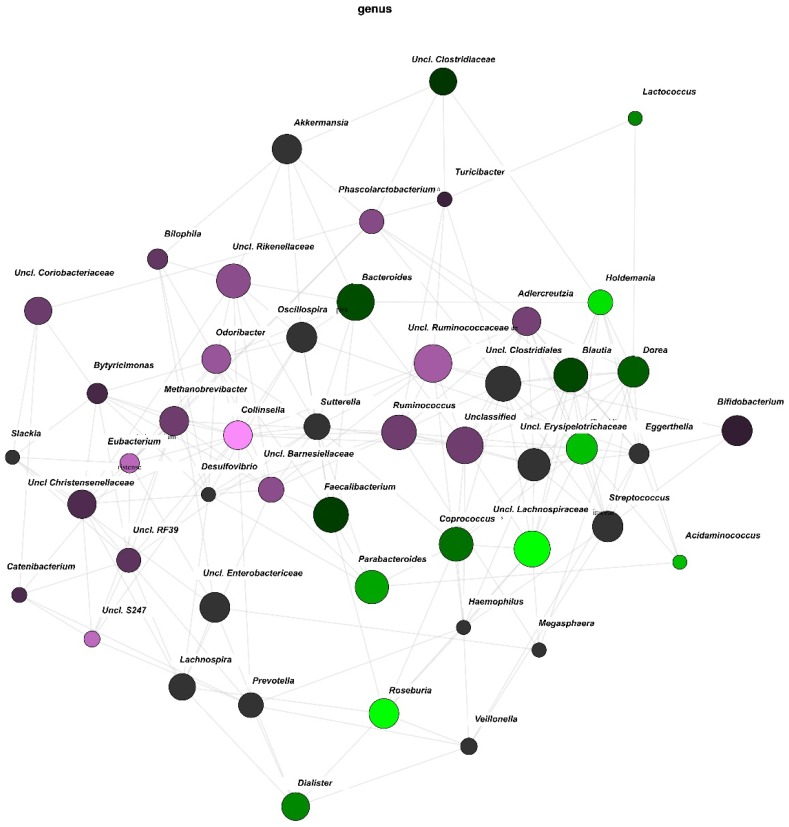
Network Analysis. The relative brightness of the nodes indicates the significance level of their association. Genera associated with an omnivorous diet are displayed in more pink/purple and those associated with a vegetarian diet are displayed in green.

Network analyses were performed to investigate if diet type altered the overall composition of the gut microbiota. A vegetarian diet in early pregnancy was associated with increased abundance of *Holdemania*, *Roseburia*, *Acidaminococcus*, *Uncl. Lachnospiraceae*, *Uncl. Erysipelotrichaceae* and *Parabacteroides*. In contrast, an omnivorous diet in early pregnancy is associated with increased abundance of *Collinsella*, *Uncl. S247*, *Ruminococcus*, and *Uncl Christensenellaceae* (Figure 3). The relative brightness of the nodes indicates the significance level of their association, highlighting the importance of *Roseburia*, *Holdemania* and *Uncl. Lachnospiraceae* in the vegetarian group and that of *Collinsella* in the omnivorous group.

### 3.4. Regression Analyses of Microbiota Profiles with Anthropometric Data

The composition of the gut microbiota was not correlated with the participants’ BMI or fasting glucose levels. Fasting circulating lipid and insulin levels as well as glycated haemoglobin (HbA1c) were correlated with abundance of specific genera (Table 3). Dietary intake of many macronutrients was correlated with gut microbiota composition (Table 3) although dietary intake of sugars, total fatty acids and mono-unsaturated fatty acids (MUFA) and total energy was not correlated to bacterial abundance. Given the observed differences in PUFA intake between the groups, this was investigated further by analysing the relationships between gut microbiota composition and dietary ω-6 and ω-3 fatty acids. The intake of the linoleic acid (ω-6) fatty acid was positively correlated with *Holdemania* (rho = 0.51, *p* = 0.006) and *Roseburia* (rho = 0.40, *p* = 0.04) abundance, but negatively correlated with *Collinsella* (rho = −0.50, *p* = 0.009), *Slackia* (rho = −0.42, *p* = 0.03) and *Uncl. Rikenellaceae* (rho = −0.40, *p* = 0.04). Arachidonic acid (ω-6) intake was positively correlated with *Bilophila* taxa (*r* = 0.44, *p* = 0.02), whereas the intake of ω-3 fatty acids eicosapentanoic acid (EPA) and docohexaenoic acid (DHA) positively correlated with an *Uncl. Rumminococcus* (*r* = 0.43, *p* = 0.02 and *r* = 0.42, *p* = 0.03 respectively); Linolenic acid (ω-3) intake was positively correlated with *Holdemania* abundance (rho = 0.44, *p* = 0.02) and negatively with *Streptococcus* (rho = −0.43, *p* = 0.02). 

### 3.5. Predicted Biosynthesis Function Analyses

**Figure 4:**
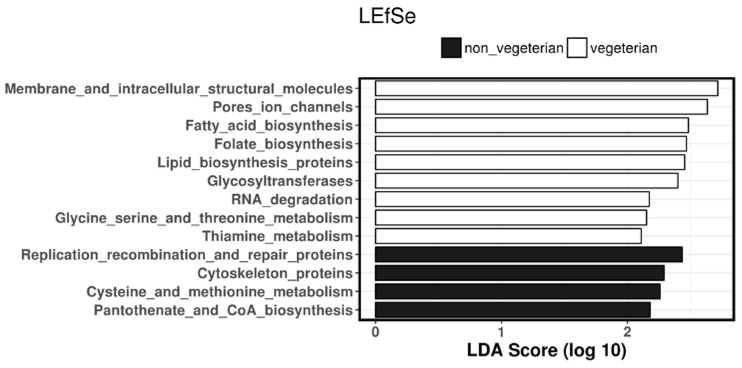
Predicted biosynthesis function analyses.

Predicted biosynthesis function analyses (Figure 4) suggested that microbiota associated with biosynthesis pathways for fatty acids, lipids, folate amongst others were more pronounced in those on vegetarian diet.

## 4. Discussion

This study explored the gut microbiota profile in women who were vegetarian or omnivorous during early gestation. There was no difference in alpha diversity, but beta diversity was reduced in vegetarians. There were differences seen in the relative abundance of several genera in those on a vegetarian diet, specifically a reduction in *Collinsella*, *Holdemania*, and an increase in *Roseburia* and *Lachnospiraceae*. Functional analyses suggested that women on a vegetarian diet had higher abundance of species involved in fatty acid and lipid synthesis.

We have previously reported a negative correlation between dietary fibre intake and *Collinsella* abundance in early pregnancy in the SPRING cohort [15]. This is consistent with the reduction in *Collinsella* seen in the current study where dietary fibre intake trended to be higher in those women who followed a vegetarian diet. *Collinsella* is positively correlated with insulin and lipid levels in the SPRING cohort [9] as well as outside pregnancy. In non-pregnant omnivorous people, *Collinsella aerofaciens* was also reported to be higher than in their vegetarian counterparts [16].

*Lachnospiraceae* abundance was positively correlated with leptin and BMI in the larger SPRING cohort. The current study found an increase in *Lachnospiraceae* in those on a vegetarian diet. *Lachnospiraceae* degrade polysaccharides to short chain fatty acids (SCFA) and in animal studies, with herbivores having a higher abundance than carnivores [17]. Outside pregnancy, a study of adults, 11 lacto-vegetarian and 20 vegans compared to 20 omnivores showed a lower abundance of *Lachnospiraceae* in those on a vegetarian diet [18].

In this study, a genus within the *Lachnospiraceae* family, *Lachnospira*, was negatively correlated with very low-density lipoprotein (VLDL) cholesterol and circulating triglycerides indicating that the associations between host factors and bacterial abundance differ between bacterial family members. The protein and sugars intake were lower in those on vegetarian than omnivorous diets, with some differences in fatty acid intake. The data from the current study supports some of the findings of other studies of the gut microbiota in pregnancy. We believe this study to be the first to examine the impact of whole dietary patterns (a vegetarian diet compared with omnivorous diet) rather than analysis based on macronutrient composition. A study of overweight and obese women at ~17 weeks gestation in Finland examined the relationship between diet composition as measured by 3-day food diary and maternal gut microbiota [19]. In this study, the analysis focused on intake of fat and fibres separating women into three groups: low fibre/moderate fat, high fibre/moderate fat and low fibre/high fat diets. *Lachnospira* was negatively correlated with VLDL particles and VLDL triglyceride content as well as overall triglyceride levels [19], similar to the negative correlation between *Lachnospira* and VLDL cholesterol levels observed in this study. Consumption of almonds, which have a relatively high proportion of PUFAs, has been shown to increase *Lachnospira*, *Roseburia*, and *Dialister* abundance in 18 healthy adults [20]. *Lachnospira* is increased in whole grain compared to refined grain diets in the setting of adults randomized to these diets for six weeks [21]. In 37 Australian children aged 2–3 years, *Lachnospira* was positively associated with vegetarian diets [22]. Lastly, *Lachnospira* abundance is decreased in overweight and obese women just after delivery suggesting that its abundance is associated with a healthy gut microbiota [23].

Members of genus *Roseburia* are Gram-positive and produce butyrate during fermentation. Butyrate is widely described as beneficial because it can increase energy uptake and utilisation by the host epithelium, as well as modulate local inflammation and cell repair via apoptosis [24]. *Roseburia* abundance is increased in the setting of the Mediterranean diet [25], suggesting that dietary fibres and carbohydrate composition have variable influence on *Roseburia* abundance [26] with resistant starches promoting microbial production of SCFA such as butyrate. In the current study, we found a greater abundance of *Roseburia* in women consuming a vegetarian diet with no difference in total intake of carbohydrate or fibre between the vegetarian and omnivore groups. However, it is possible that with a larger cohort size, women on a vegetarian diet would have higher dietary fibre intake given that the median intake tended to be higher in women on a vegetarian diet in this study. A positive correlation between *Roseburia* and fibre intake is commonly reported and seen in the current study as well. Even though fibre intake did not differ, the type of fibre in the diet of those in our study may have differed between groups, which could explain the difference in *Roseburia* abundance. *Roseburia* abundance was also positively correlated with PUFA intake in this study. PUFA intake was higher in women on a vegetarian diet especially of ω-6 fatty acids such as linoleic acid. Linoleic acid was also positively correlated with *Roseburia* abundance. Given that some Gram-positive bacteria (such as *Roseburia*) can use exogenous fatty acids for the biosynthesis of lipids and fatty acids [27], it is possible that the higher abundance of PUFAs drives the higher abundance of fatty acid biosynthesis pathways. *Roseburia* is found in the mucosal part of the gut microbiota rather than the luminal part and there is some evidence that it may serve to protect other mucosal bacteria such as *Faecalibacterium prausnitzii* from the detrimental effects of linoleic acid through biohydrogenating the linoleic acid to stearic acid [28]. A small randomised controlled trial of a ω-3 PUFA in healthy middle-aged individuals increased *Roseburia* abundance when given conjugated to triglycerides but as ethyl ester conjugates [29] indicating that the form in which PUFAs are presented to the gut microbiota may be of importance to their effects.

*Holdemania* is a Gram-positive anaerobic genus from the family *Erysipelotrichaceae*. We saw an increased abundance of *Holdemania* and *Uncl.Erysipelotrichaceae* in women on vegetarian diet. *Holdemania* abundance was positively correlated with intake of dietary fibre, overall PUFA and both ω-3 and ω-6 PUFAs in this study. We have previously reported that in network analyses of pregnant women with high versus low dietary fibre intake, high fibre intake is associated with higher abundance of *Holdemania* [15]. *Holdemania* abundance has been reported to increase with dietary resistant maltodextrin diets in healthy adult men [30]. It may be that *Holdemania*, which is saccharolytic and does not grow on animal protein, is emblematic for a diet low in animal protein [31].

The strength of the current study is in the carefully characterised and detailed data on the participants. Our results are also internally consistent with the findings from the larger SPRING cohort around fibre intake. The overall numbers included in these sub-analyses are small, and many of the findings here need to be explored in larger cohorts. Our study provides valuable preliminary data to provide insight into some of the factors driving differences in maternal metabolism in pregnancy.

## 5. Conclusions

This study suggests that a vegetarian diet in early pregnancy is associated with a different composition of the gut microbiota compared to an omnivorous diet. A vegetarian diet is associated with higher abundance of bacteria that produce SCFA. It is unclear if this results in higher circulating SCFA, a healthier gut mucosa and lower levels of inflammation.

## Figures and Tables

**Table 1:** Participant characteristics.

	Vegetarian	Omnivorous	*p* Value
*N*	9	18	ND
Maternal age (years)	33 (29–34)	34 (32–37)	0.38
Maternal BMI (kg/m^2^) *	28.3 (26.5–35.5)	28.4 (26.5–35–3)	0.91
Ethnicity			ND
Caucasian (%)	7 (77.8)	16 (88.8)	
Indian (%)	2 (22.2)	1 (5.6)	
Asian (%)	0 (0)	1 (5.6)	
Parity ^$^	0 (0–2)	1 (1–2)	ND
Systolic blood pressure (mmHg)	110 (107–118)	110 (101–112)	0.46
Diastolic blood pressure (mmHg)	60 (58–70)	63 (60–70)	0.59
Glucose (mmol/L)	4.5 (4.4–4.6)	4.3 (4.1–4.4)	**0.04**
HbA1c (%)	4.8 (4.7–5.1)	4.7 (4.6–5.0)	0.41
Future GDM (%)	1 (11.1)	2 (11.1)	1
Insulin	4.7 (3.8–7.4)	7.3 (4.6–8.3)	0.38
Total Cholesterol (mmol/L)	5.4 (4.7–6.1)	5.3 (4.6–5.9)	0.71
HDL cholesterol (mmol/L)	2 (1.7–2.2)	1.7 (1.6–2.0)	0.11
LDL cholesterol (mmol/L)	3 (2.5–3.2)	3.0 (2.4–3.3)	0.88
VLDL cholesterol (mmol/L)	0.6 (0.4–0.7)	0.6 (0.4–0.8)	0.75
Triglycerides (mmol/L)	1.3 (1.0–1.8)	1.4 (0.8–1.7)	0.87
Fetal sex (F/M)	6/3	11/7	1
Birth weight (g)	3572 (3193–3992)	3397 (2976–3978)	0.52
Birth length (cm)	51.5 (49.1–53.0)	50 (49.2–55.3)	0.82

All data are presented as median (IQR), comparisons between the groups were made by Mann–Whitney *U* testing. All circulating metabolic markers were determined fasting. * determined at study entry; ^$^ data missing from five vegetarians and four controls. ND: not-determined; BMI: body mass index; GDM: gestational diabetes mellitus; HDL: high-density lipoprotein; LDL: low-densitiy lipoprotein; VLDL: very low-density lipoprotein.

**Table 2:** Dietary characteristics.

	Vegetarian	Omnivorous	*p* Value
Overall Energy intake (kJ/day)	4988 (3531–6387)	5528 (5021–6428)	0.15
Protein (g/day)	42.5 (32.7–59.2)	71.1 (61.3–77)	**0.0003**
Iron intake (g/day))	8.1	8.4	0.56
Carbohydrate (g/day)	129.1 (106.5–154.4)	144.6 (133.5–167)	0.21
Starch (g/day)	76.5 (47.5–95.1)	74.0 (54.1–88.0)	0.78
Sugars (g/day)	56.0 (44.2–72.1)	75.3 (64.2–92.3)	**0.02**
Dietary fibre (g/day)	20.9 (13.9–26.7)	16.5 (13.5–19.6)	0.13
Glycaemic Index	51.5 (49.0–52.1)	49.1 (46.7–52.6)	0.40
Saturated fatty acids (g/day)	13.9 (11.3–23.8)	21.5 (17.4–26.5)	**0.04**
Monounsaturated fatty acids (g/day)	18.0 (10.6–23.8)	18.9 (16.4–23.5)	0.53
Polyunsaturated fatty acids (g/day)	11.6 (9.1–15.8)	6.5 (5.0–8.6)	**0.006**
α-Linoleic acid (g/day)	0.97	0.63	0.16
Linoleic acid (g/day)	10.4	5.7	**0.008**
Arachidonic acid (g/day)	0.02	0.05	**0.05**
EPA (g/day)	0.003	0.078	**0.002**
DHA (g/day)	0.01	0.17	**0.002**

All data is presented as median (IQR), comparisons between the groups were made by Mann–Whitney *U* testing. EPA: eicosapentanoic acid; DHA: docosahexanoic acid.

**Table 3:** Correlations between specific genera and clinical characteristics and dietary intake.

	Genus	Rho	*p* Value
HbA1c	*Ruminococcus*	−0.59	0.002
	*Turicibacter*	−0.47	0.016
Insulin	*Coprococcus*	−0.38	0.050
Total cholesterol	*Uncl. RF39*	−0.40	0.036
	*Ruminococcus*	0.39	0.043
HDL cholesterol	*Uncl. Coriobacteriaceae*	−0.43	0.027
	*Parabacteroides*	0.38	0.050
LDL cholesterol	*Uncl. RF39*	−0.43	0.029
VLDL cholesterol	*Lachnospira*	−0.43	0.029
	*Collinsella*	0.39	0.048
Triglycerides	*Lachnospira*	−0.40	0.038
**Dietary Intake (g/day)**			
Protein	*Adlercreutzia*	0.46	0.017
Carbohydrates	*Dialister*	−0.42	0.028
	*Ruminococcus*	0.41	0.034
Starch	*Dialister*	−0.47	0.013
	*Uncl. Rikenellaceae*	−0.42	0.030
	*Coprococcus*	0.41	0.033
	*Uncl. Clostridiaceae*	0.39	0.047
Fibre	*Uncl. Lachnospiraceae*	0.67	0.0002
	*Coprococcus*	0.58	0.002
	*Haemophilus*	0.44	0.021
	*Roseburia*	0.42	0.031
	*Clostridium*	0.41	0.036
	*Holdemania*	0.38	0.050
Glycaemic index	*Holdemania*	0.51	0.007
	*Prevotella*	−0.48	0.011
	*Uncl. Costridiaceae*	0.44	0.020
Poly-unsaturated fatty acids	*Holdemania*	0.47	0.012
	*Collinsella*	−0.46	0.017
	*Roseburia*	0.41	0.033
	*Uncl. Rikenellaceae*	−0.41	0.035
Saturated fatty acids	*Roseburia*	−0.40	0.038

## Supplementary Materials

The following are available online at http://www.mdpi.com/2072-6643/10/7/890/s1, Figure S1: Alpha diversity analysis on Phylum level, Figure S2: Alpha diversity analysis on Genus level, Figure S3: Alpha diversity analysis on OTU level, Figure S4: Beta diversity analysis on OTU level, Figure S5: (A) Group comparison (B) LEfSe analysis on family level, Figure S6: Group comparison on OTU level, Figure S7: Group comparison on OTU level.
